# Resection of the Inferior Maxillary in a Case of Cancer of the Bone

**Published:** 1859-01

**Authors:** 


### ARTICLE VI.

*Resection of the Inferior Maxillary in a case of Cancer of the Bone.* Translated for the American Journal of Dental Science.

On the 8th of June we were called upon to see Reverend Joseph Carvalho, a bachelor, proprietor, 54 years of age, of good constitution and sanguine temperament, in company with MM. Arante and Magalhaès Continho. We became convinced of the existence of a tumor of the size of a hen's egg, the greater part of which was hard, like cartilage and the other part soft, fungous, adhering to the posterior part of the dental arch and stretching underneath the tongue to the base of that organ and which we were able to examine completely with our fingers. But a month and a half had transpired since Carvalho became aware of its development; since then it has increased gradually in size, but without causing the least pain and not compromising either the glands or the lymphatic ganglions of the neighboring regions. The tumor stretched from the inferior dental arch to which it was attached, to an inch and a half underneath the tongue. Laterally, it was limited on the left by the three molar teeth, on the right by the canine teeth. The anterior part had not become altered, either in form or consistence. The two incisors of the left side and the first incisor of the right side had been loosened. The hard part, which stretched underneath the tongue, and which was discovered first, formed the greater part of this tumor. The fungous portion resembled an appendage of the size of a large olive placed near the dental arch. It had been the seat of several abundant hemorrhages.

From the consultation, we came to a unanimous opinion; we diagnosticated a cancer; but we were unable to determine whether the inferior maxillary was the primitive seat of the disease or whether it made its appearance there subsequently. Notwithstanding, we thought that the only chance of cure was to extirpate the tumor with part of the bone upon which it was found, and that the patient should go to the hospital of Saint Joseph or to the *ma son deisantê* where he would receive better care than at home.

The patient preferred to lodge in a private part of the hospital and on the 9th of June he was again examined by the physicians who had been called in consultation and also by many other physicians of the hospital, among whom were MM. Barral, Sibeiro, Viana, Arnaut, Theotonio de Silva, May Fiqueiro, Santos, Avellar, etc. ; all of these practitioners were present during the operation which M. Magalhaès Continho performed the next day in the following manner : after having extracted the right canine and the third left molar, a vertical incision was made along the middle of the lower lip, then, after having separated the two fragments from the base of the bone, the bone was resected with the chain saw through the spaces made by the extracted teeth. At length after having tied the tongue with a thread passed through its base by means of a curved needle, (to prevent the inconveniences which might result from the severing of the genio-glosse) we removed the tumor with a portion of the bone to which it adhered. We were obliged to tie three small arteries which bled a little ; the integuments were re-united along the median line with four sutures, and the tongue was maintained in its place by the means we have mentioned. The appropriate bandages were then at the proper time put on. The dissection of the pathological specimen taken away and the examination under the microscope, by our celebrated micrographer, Dr. May Fiquiero confirmed our diagnosis, and we found that the bone had been the primitive seat of the disease. At this time the patient is in a satisfactory state.

[*L'Art Dentaire.*

